# Understanding the Patient Experience Through the Lenses of Racial/Ethnic and Gender Patient-Physician Concordance

**DOI:** 10.1001/jamanetworkopen.2020.25349

**Published:** 2020-11-02

**Authors:** Antoinette Schoenthaler, Joseph Ravenell

**Affiliations:** Center for Healthful Behavior Change, Department of Population Health, New York University Grossman School of Medicine, New York; Center for Healthful Behavior Change, Department of Population Health, New York University Grossman School of Medicine, New York; Office of Diversity and Inclusion, New York University Grossman School of Medicine, New York

The quality of the patient-physician relationship plays an influential role on patient outcomes. The race/ethnicity and gender of the patient and the physician are 2 aspects of the relationship that have emerged as potential reasons for the differential health outcomes experienced by some patients. While several studies have documented positive associations between patient outcomes and race-concordant and gender-concordant relationships (ie, when patient and physician are of the same racial/ethnic or gender background), a 2009 study^1^ found no such association. The study by Takeshita et al^2^ aims to address the mixed evidence base by evaluating the associations of racial/ethnic and gender concordance with patient experience in a diverse, urban network of ambulatory care practices. This cross-sectional study included 92 238 unique patients and 747 physicians across 4 racial/ethnic groups (ie, White, Black, Asian, and Hispanic) and male or female gender. Patient experience was evaluated as the likelihood of recommending the patient’s physician to others on a scale, derived from the Press Ganey Outpatient Medical Practice Survey, of 1 (very poor) to 5 (very good). The associations between patient experience and contextual factors at the level of the patient, physician, and clinical encounter were also examined.

Overall, most patients reported high ratings for their physicians. In 67 290 gender-concordant patient-physician clinical encounters, 57 924 physicians (86.1%) received the maximum score, while in 50 299 gender-discordant encounters, 42 860 physicians (85.2%) received the maximum score, with no significant differences between these groups. In 77 051 race/ethnic-concordant encounters, 67 504 physicians (87.6%) received the maximum score, while in 40 538 race/ethnic-discordant encounters, 33 280 physicians (82.1%) received the maximum score. Despite these high ratings, several key findings emerged that differentiated patient-physician encounters by race/ethnicity. Asian patients were least likely to give high ratings of their experiences (mean [SD] score, 4.61 [0.78]), regardless of the race/ethnicity of the physician. White patients gave the highest ratings for all physicians (mean [SD] score, 4.80 [0.63]). Compared with patients in race-concordant relationships, White and Black patients were significantly more likely to give lower ratings in race-discordant relationships with Asian physicians, and Black patients were significantly more likely to give lower ratings to White physicians. There were no significant associations between patient ratings and racial/ethnic discordance for any other race/ethnicity-discordant relationship. Black and Asian race, higher education levels, and primary language other than English were 3 patient characteristics associated with lower odds of giving the highest score to physicians. Older physician age was associated with lower odds of receiving the highest score, as were clinical encounters in specialties other than internal or family medicine (eg, surgical medicine).

This study has several limitations, which are reflective of the current literature. Among 92 238 patients, 75 307 individuals (81.6%) self-identified as White, and among 747 physicians, 533 individuals (71.4%) self-identified as White. Most patients and physicians reported higher levels of socioeconomic status. Thus, these studies still fail to reach the patient populations with the highest risk of receiving unequal care. The survey had an approximately 20% response rate, and only 1 survey was included for each patient-physician pair. A single survey of a longitudinal patient-physician relationship is often not reflective of the ongoing relationship. Patient responses were also highly skewed, with most scores at the maximum level.

Nonetheless, these findings have extremely timely and critical implications for the delivery of care in an increasingly racially and ethnically diverse society. As noted by the investigators, greater investment is needed in recruiting, mentoring, and retaining medical students, residents, and practicing physicians who identify as members of underrepresented minority groups. Training in principles of cultural humility and multiculturalism, which emphasize a lifelong commitment to recognizing one’s unique perspective and experiences and addressing biases toward others, should be mandatory for physicians of all ages, genders, and race/ethnic backgrounds.^3^ It is also vital to receive training in structural competency, that is, the ability of physicians and trainees to appreciate how social determinants of health can influence symptoms, diseases, and attitudes toward patients, populations, and health systems.^4^ These trainings are paramount in bridging the gaps in cross-cultural and cross-racial communication that are associated with outcomes in race-discordant patient-physician relationships. However, the clinical encounter is a dyadic process; patients and physicians influence each other’s thoughts, attitudes, emotions, and behaviors in a reciprocal way. Failure to also address the stereotypes patients hold about physicians’ knowledge and capabilities based on the physician’s race/ethnicity and place of birth limits the potential to create collaborative relationships that lead to better patient outcomes.^5^ Because of this, it is vital that patients also receive training opportunities in implicit bias. Additionally, promoting positive images of members of underrepresented minority groups in society and promoting diverse representations of “what doctors look like” remain critical strategies for eradicating stereotypes that prevent patients from viewing physicians who are members of underrepresented minority groups as trustworthy and competent. Calls to action for trainings that promote the delivery of equitable care are as urgent as ever. However, they will fall short if the sociopolitical climate continues to devalue racial/ethnic diversity and if policies that penalize institutions for engaging in academic activities to address systemic racism, promote equity, and embrace antiracism are allowed to stand.^6^
